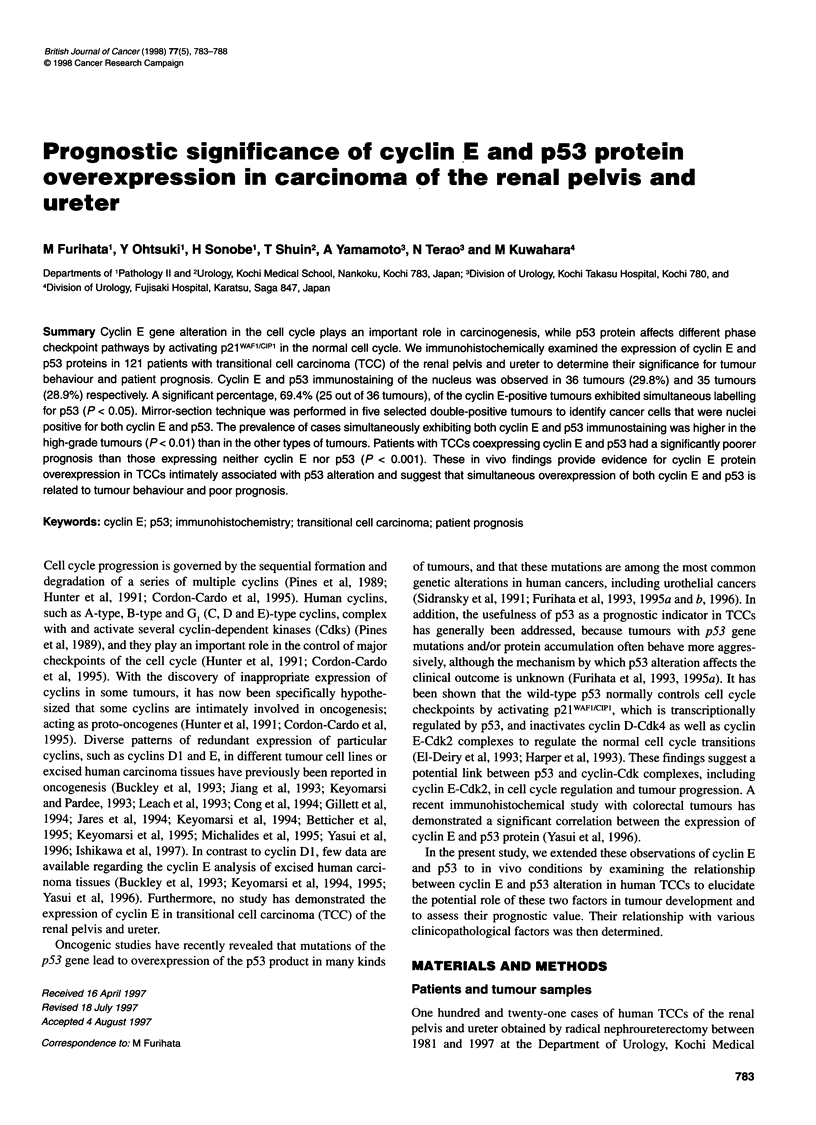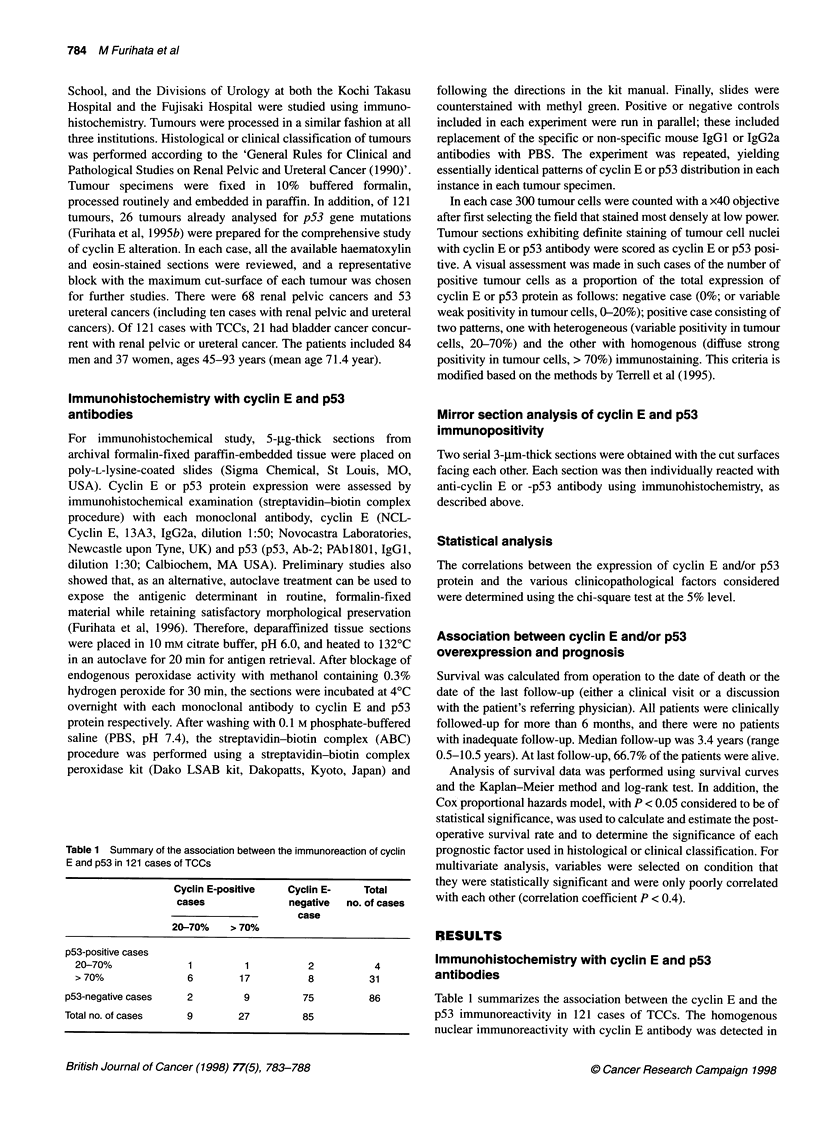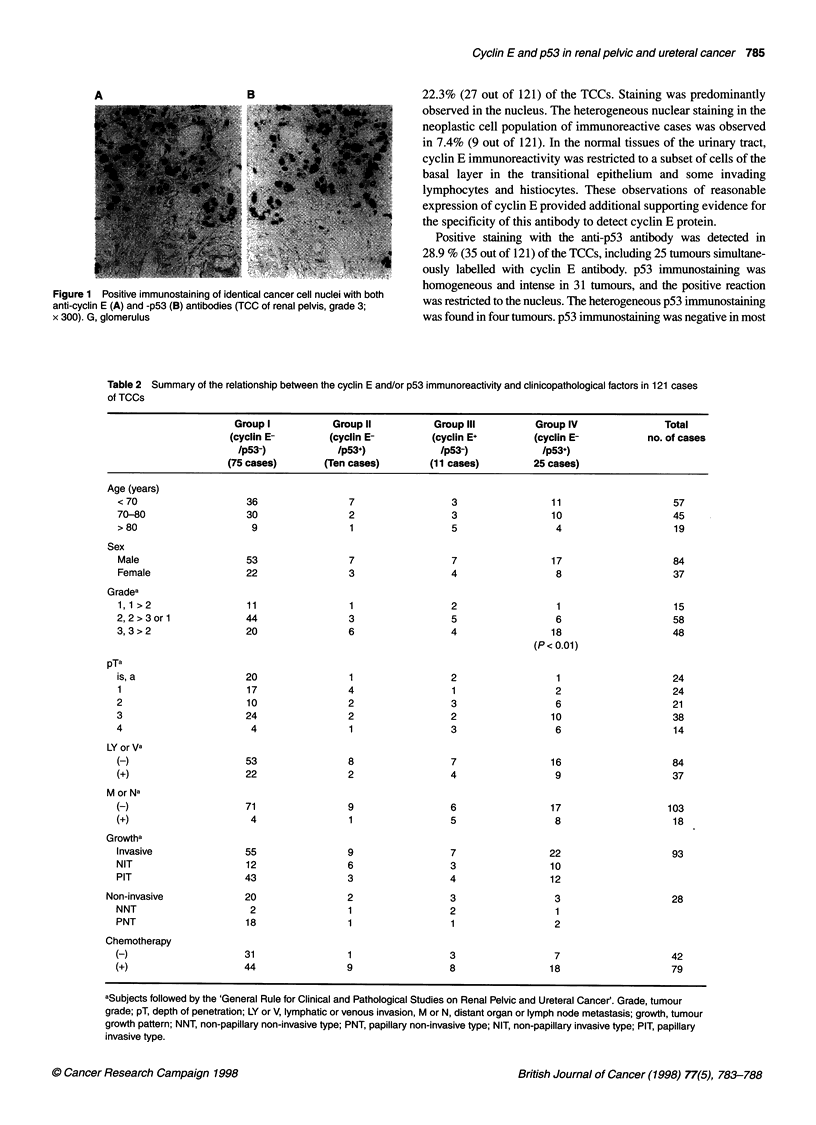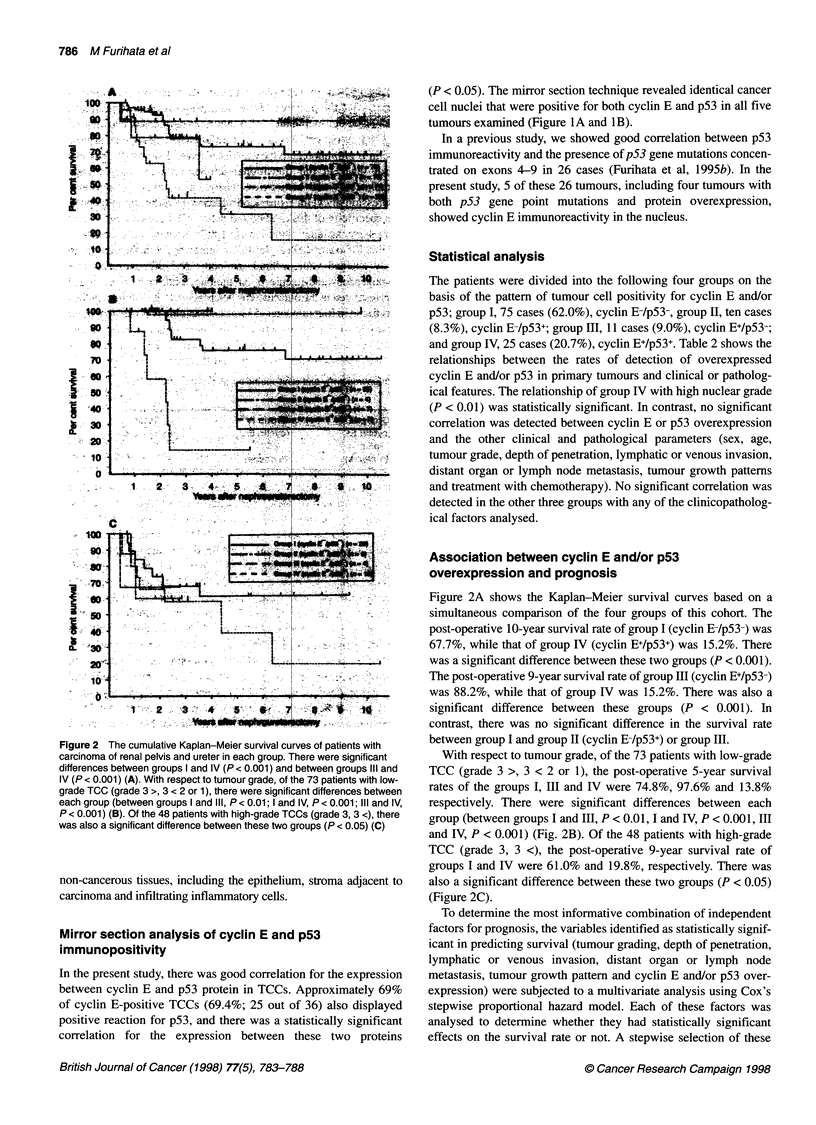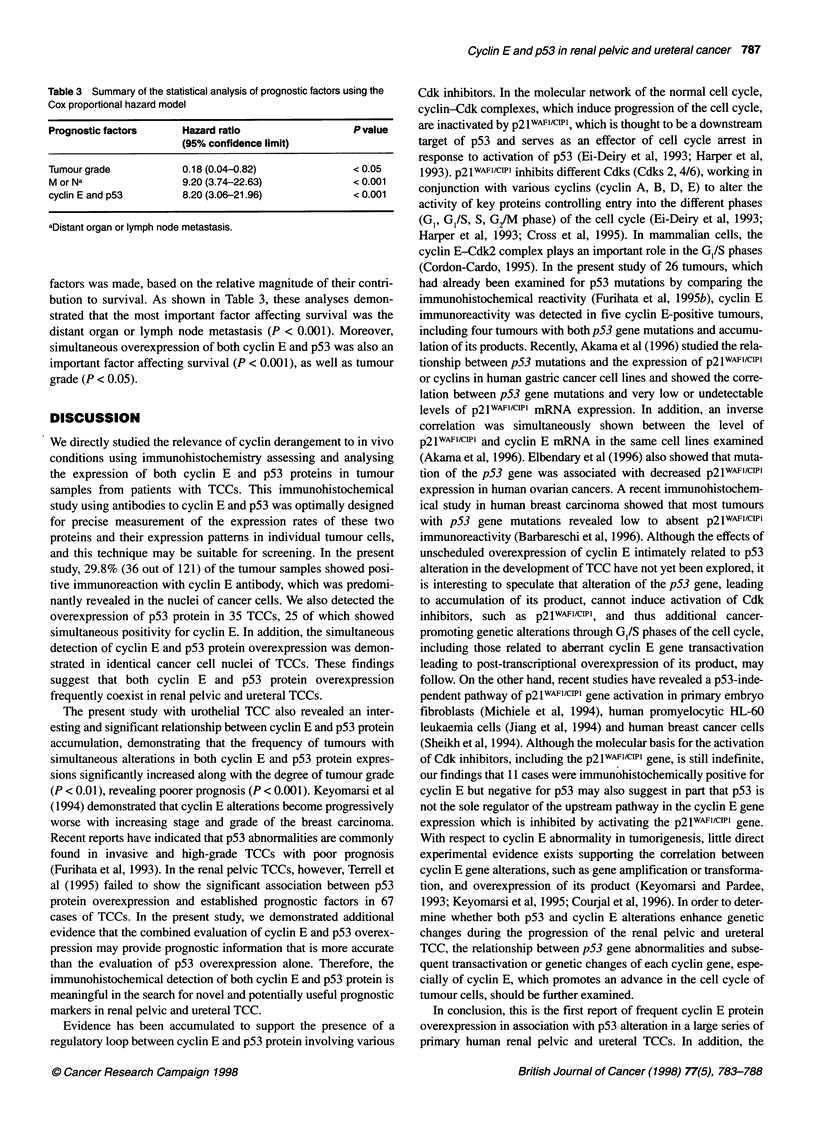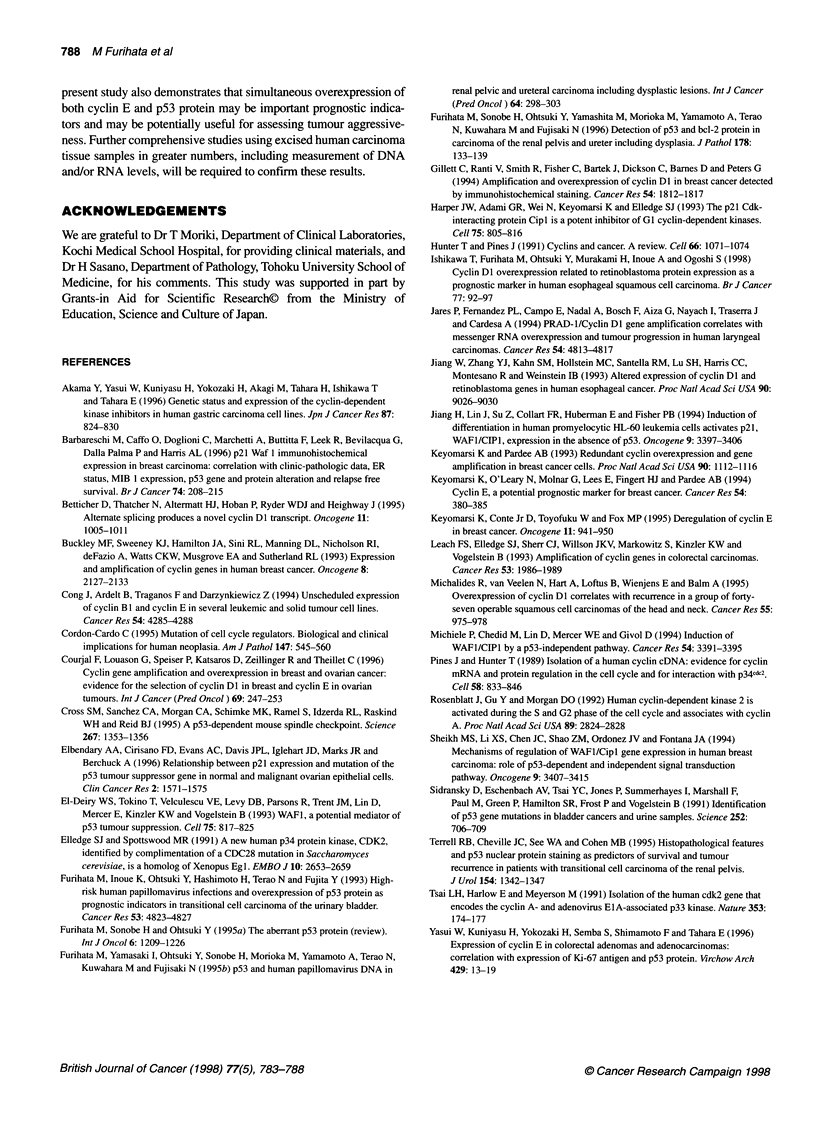# Prognostic significance of cyclin E and p53 protein overexpression in carcinoma of the renal pelvis and ureter.

**DOI:** 10.1038/bjc.1998.127

**Published:** 1998-03

**Authors:** M. Furihata, Y. Ohtsuki, H. Sonobe, T. Shuin, A. Yamamoto, N. Terao, M. Kuwahara

**Affiliations:** Department of Pathology II, Kochi Medical School, Nankoku, Japan.

## Abstract

**Images:**


British Journal of Cancer (1998) 77(5), 783–788
© 1998 Cancer Research Campaign

### Prognostic significance of cyclin E and p53 protein overexpression in carcinoma of the renal pelvis and ureter

**M Furihata[1], Y Ohtsuki[1], H Sonobe[1], T Shuin[2], A Yamamoto[3], N Terao[3] and M Kuwahara[4]**

Departments of [1]Pathology II and [2]Urology, Kochi Medical School, Nankoku, Kochi 783, Japan; [3]Division of Urology, Kochi Takasu Hospital, Kochi 780, and [4]Division of Urology, Fujisaki Hospital, Karatsu, Saga 847, Japan

**Summary** Cyclin E gene alteration in the cell cycle plays an important role in carcinogenesis, while p53 protein affects different phase checkpoint pathways by activating p21$^{WAF1/CIP1}$ in the normal cell cycle. We immunohistochemically examined the expression of cyclin E and p53 proteins in 121 patients with transitional cell carcinoma (TCC) of the renal pelvis and ureter to determine their significance for tumour behaviour and patient prognosis. Cyclin E and p53 immunostaining of the nucleus was observed in 36 tumours (29.8%) and 35 tumours (28.9%) respectively. A significant percentage, 69.4% (25 out of 36 tumours), of the cyclin E-positive tumours exhibited simultaneous labelling for p53 ($P < 0.05$). Mirror-section technique was performed in five selected double-positive tumours to identify cancer cells that were nuclei positive for both cyclin E and p53. The prevalence of cases simultaneously exhibiting both cyclin E and p53 immunostaining was higher in the high-grade tumours ($P < 0.01$) than in the other types of tumours. Patients with TCCs coexpressing cyclin E and p53 had a significantly poorer prognosis than those expressing neither cyclin E nor p53 ($P < 0.001$). These in vivo findings provide evidence for cyclin E protein overexpression in TCCs intimately associated with p53 alteration and suggest that simultaneous overexpression of both cyclin E and p53 is related to tumour behaviour and poor prognosis.

**Keywords:** cyclin E; p53; immunohistochemistry; transitional cell carcinoma; patient prognosis

Cell cycle progression is governed by the sequential formation and degradation of a series of multiple cyclins (Pines et al, 1989; Hunter et al, 1991; Cordon-Cardo et al, 1995). Human cyclins, such as A-type, B-type and $G_1$ (C, D and E)-type cyclins, complex with and activate several cyclin-dependent kinases (Cdks) (Pines et al, 1989), and they play an important role in the control of major checkpoints of the cell cycle (Hunter et al, 1991; Cordon-Cardo et al, 1995). With the discovery of inappropriate expression of cyclins in some tumours, it has now been specifically hypothesized that some cyclins are intimately involved in oncogenesis; acting as proto-oncogenes (Hunter et al, 1991; Cordon-Cardo et al, 1995). Diverse patterns of redundant expression of particular cyclins, such as cyclins D1 and E, in different tumour cell lines or excised human carcinoma tissues have previously been reported in oncogenesis (Buckley et al, 1993; Jiang et al, 1993; Keyomarsi and Pardee, 1993; Leach et al, 1993; Cong et al, 1994; Gillett et al, 1994; Jares et al, 1994; Keyomarsi et al, 1994; Betticher et al, 1995; Keyomarsi et al, 1995; Michalides et al, 1995; Yasui et al, 1996; Ishikawa et al, 1997). In contrast to cyclin D1, few data are available regarding the cyclin E analysis of excised human carcinoma tissues (Buckley et al, 1993; Keyomarsi et al, 1994, 1995; Yasui et al, 1996). Furthermore, no study has demonstrated the expression of cyclin E in transitional cell carcinoma (TCC) of the renal pelvis and ureter.

Oncogenic studies have recently revealed that mutations of the *p53* gene lead to overexpression of the p53 product in many kinds of tumours, and that these mutations are among the most common genetic alterations in human cancers, including urothelial cancers (Sidransky et al, 1991; Furihata et al, 1993, 1995*a* and *b*, 1996). In addition, the usefulness of p53 as a prognostic indicator in TCCs has generally been addressed, because tumours with *p53* gene mutations and/or protein accumulation often behave more aggressively, although the mechanism by which p53 alteration affects the clinical outcome is unknown (Furihata et al, 1993, 1995*a*). It has been shown that the wild-type p53 normally controls cell cycle checkpoints by activating p21$^{WAF1/CIP1}$, which is transcriptionally regulated by p53, and inactivates cyclin D-Cdk4 as well as cyclin E-Cdk2 complexes to regulate the normal cell cycle transitions (El-Deiry et al, 1993; Harper et al, 1993). These findings suggest a potential link between p53 and cyclin-Cdk complexes, including cyclin E-Cdk2, in cell cycle regulation and tumour progression. A recent immunohistochemical study with colorectal tumours has demonstrated a significant correlation between the expression of cyclin E and p53 protein (Yasui et al, 1996).

In the present study, we extended these observations of cyclin E and p53 to in vivo conditions by examining the relationship between cyclin E and p53 alteration in human TCCs to elucidate the potential role of these two factors in tumour development and to assess their prognostic value. Their relationship with various clinicopathological factors was then determined.

### MATERIALS AND METHODS

### Patients and tumour samples

One hundred and twenty-one cases of human TCCs of the renal pelvis and ureter obtained by radical nephroureterectomy between 1981 and 1997 at the Department of Urology, Kochi Medical

*Received 16 April 1997*
*Revised 18 July 1997*
*Accepted 4 August 1997*

*Correspondence to:* M Furihata

School, and the Divisions of Urology at both the Kochi Takasu Hospital and the Fujisaki Hospital were studied using immunohistochemistry. Tumours were processed in a similar fashion at all three institutions. Histological or clinical classification of tumours was performed according to the 'General Rules for Clinical and Pathological Studies on Renal Pelvic and Ureteral Cancer (1990)'. Tumour specimens were fixed in 10% buffered formalin, processed routinely and embedded in paraffin. In addition, of 121 tumours, 26 tumours already analysed for *p53* gene mutations (Furihata et al, 1995*b*) were prepared for the comprehensive study of cyclin E alteration. In each case, all the available haematoxylin and eosin-stained sections were reviewed, and a representative block with the maximum cut-surface of each tumour was chosen for further studies. There were 68 renal pelvic cancers and 53 ureteral cancers (including ten cases with renal pelvic and ureteral cancers). Of 121 cases with TCCs, 21 had bladder cancer concurrent with renal pelvic or ureteral cancer. The patients included 84 men and 37 women, ages 45–93 years (mean age 71.4 year).

### Immunohistochemistry with cyclin E and p53 antibodies

For immunohistochemical study, 5-μg-thick sections from archival formalin-fixed paraffin-embedded tissue were placed on poly-L-lysine-coated slides (Sigma Chemical, St Louis, MO, USA). Cyclin E or p53 protein expression were assessed by immunohistochemical examination (streptavidin–biotin complex procedure) with each monoclonal antibody, cyclin E (NCL-Cyclin E, 13A3, IgG2a, dilution 1:50; Novocastra Laboratories, Newcastle upon Tyne, UK) and p53 (p53, Ab-2; PAb1801, IgG1, dilution 1:30; Calbiochem, MA USA). Preliminary studies also showed that, as an alternative, autoclave treatment can be used to expose the antigenic determinant in routine, formalin-fixed material while retaining satisfactory morphological preservation (Furihata et al, 1996). Therefore, deparaffinized tissue sections were placed in 10 mM citrate buffer, pH 6.0, and heated to 132°C in an autoclave for 20 min for antigen retrieval. After blockage of endogenous peroxidase activity with methanol containing 0.3% hydrogen peroxide for 30 min, the sections were incubated at 4°C overnight with each monoclonal antibody to cyclin E and p53 protein respectively. After washing with 0.1 M phosphate-buffered saline (PBS, pH 7.4), the streptavidin–biotin complex (ABC) procedure was performed using a streptavidin–biotin complex peroxidase kit (Dako LSAB kit, Dakopatts, Kyoto, Japan) and following the directions in the kit manual. Finally, slides were counterstained with methyl green. Positive or negative controls included in each experiment were run in parallel; these included replacement of the specific or non-specific mouse IgG1 or IgG2a antibodies with PBS. The experiment was repeated, yielding essentially identical patterns of cyclin E or p53 distribution in each instance in each tumour specimen.

In each case 300 tumour cells were counted with a ×40 objective after first selecting the field that stained most densely at low power. Tumour sections exhibiting definite staining of tumour cell nuclei with cyclin E or p53 antibody were scored as cyclin E or p53 positive. A visual assessment was made in such cases of the number of positive tumour cells as a proportion of the total expression of cyclin E or p53 protein as follows: negative case (0%; or variable weak positivity in tumour cells, 0–20%); positive case consisting of two patterns, one with heterogeneous (variable positivity in tumour cells, 20–70%) and the other with homogenous (diffuse strong positivity in tumour cells, > 70%) immunostaining. This criteria is modified based on the methods by Terrell et al (1995).

**Table 1** Summary of the association between the immunoreaction of cyclin E and p53 in 121 cases of TCCs

| | Cyclin E-positive cases | | Cyclin E-negative case | Total no. of cases |
|---|---|---|---|---|
| | 20–70% | > 70% | | |
| p53-positive cases | | | | |
| 20–70% | 1 | 1 | 2 | 4 |
| > 70% | 6 | 17 | 8 | 31 |
| p53-negative cases | 2 | 9 | 75 | 86 |
| Total no. of cases | 9 | 27 | 85 | |

### Mirror section analysis of cyclin E and p53 immunopositivity

Two serial 3-μm-thick sections were obtained with the cut surfaces facing each other. Each section was then individually reacted with anti-cyclin E or -p53 antibody using immunohistochemistry, as described above.

### Statistical analysis

The correlations between the expression of cyclin E and/or p53 protein and the various clinicopathological factors considered were determined using the chi-square test at the 5% level.

### Association between cyclin E and/or p53 overexpression and prognosis

Survival was calculated from operation to the date of death or the date of the last follow-up (either a clinical visit or a discussion with the patient's referring physician). All patients were clinically followed-up for more than 6 months, and there were no patients with inadequate follow-up. Median follow-up was 3.4 years (range 0.5–10.5 years). At last follow-up, 66.7% of the patients were alive.

Analysis of survival data was performed using survival curves and the Kaplan–Meier method and log-rank test. In addition, the Cox proportional hazards model, with $P < 0.05$ considered to be of statistical significance, was used to calculate and estimate the post-operative survival rate and to determine the significance of each prognostic factor used in histological or clinical classification. For multivariate analysis, variables were selected on condition that they were statistically significant and were only poorly correlated with each other (correlation coefficient $P < 0.4$).

### RESULTS

### Immunohistochemistry with cyclin E and p53 antibodies

Table 1 summarizes the association between the cyclin E and the p53 immunoreactivity in 121 cases of TCCs. The homogenous nuclear immunoreactivity with cyclin E antibody was detected in

© Cancer Research Campaign 1998

**Figure 1** Positive immunostaining of identical cancer cell nuclei with both anti-cyclin E (**A**) and -p53 (**B**) antibodies (TCC of renal pelvis, grade 3; × 300). G, glomerulus

22.3% (27 out of 121) of the TCCs. Staining was predominantly observed in the nucleus. The heterogeneous nuclear staining in the neoplastic cell population of immunoreactive cases was observed in 7.4% (9 out of 121). In the normal tissues of the urinary tract, cyclin E immunoreactivity was restricted to a subset of cells of the basal layer in the transitional epithelium and some invading lymphocytes and histiocytes. These observations of reasonable expression of cyclin E provided additional supporting evidence for the specificity of this antibody to detect cyclin E protein.

Positive staining with the anti-p53 antibody was detected in 28.9 % (35 out of 121) of the TCCs, including 25 tumours simultaneously labelled with cyclin E antibody. p53 immunostaining was homogeneous and intense in 31 tumours, and the positive reaction was restricted to the nucleus. The heterogeneous p53 immunostaining was found in four tumours. p53 immunostaining was negative in most

**Table 2** Summary of the relationship between the cyclin E and/or p53 immunoreactivity and clinicopathological factors in 121 cases of TCCs

| | Group I (cyclin E$^-$/p53$^-$) (75 cases) | Group II (cyclin E$^-$/p53$^+$) (Ten cases) | Group III (cyclin E$^+$/p53$^-$) (11 cases) | Group IV (cyclin E$^-$/p53$^+$) 25 cases) | Total no. of cases |
|---|---|---|---|---|---|
| Age (years) | | | | | |
| < 70 | 36 | 7 | 3 | 11 | 57 |
| 70–80 | 30 | 2 | 3 | 10 | 45 |
| > 80 | 9 | 1 | 5 | 4 | 19 |
| Sex | | | | | |
| Male | 53 | 7 | 7 | 17 | 84 |
| Female | 22 | 3 | 4 | 8 | 37 |
| Grade[a] | | | | | |
| 1, 1 > 2 | 11 | 1 | 2 | 1 | 15 |
| 2, 2 > 3 or 1 | 44 | 3 | 5 | 6 | 58 |
| 3, 3 > 2 | 20 | 6 | 4 | 18 | 48 |
| | | | | ($P$ < 0.01) | |
| pT[a] | | | | | |
| is, a | 20 | 1 | 2 | 1 | 24 |
| 1 | 17 | 4 | 1 | 2 | 24 |
| 2 | 10 | 2 | 3 | 6 | 21 |
| 3 | 24 | 2 | 2 | 10 | 38 |
| 4 | 4 | 1 | 3 | 6 | 14 |
| LY or V[a] | | | | | |
| (–) | 53 | 8 | 7 | 16 | 84 |
| (+) | 22 | 2 | 4 | 9 | 37 |
| M or N[a] | | | | | |
| (–) | 71 | 9 | 6 | 17 | 103 |
| (+) | 4 | 1 | 5 | 8 | 18 |
| Growth[a] | | | | | |
| Invasive | 55 | 9 | 7 | 22 | 93 |
| NIT | 12 | 6 | 3 | 10 | |
| PIT | 43 | 3 | 4 | 12 | |
| Non-invasive | 20 | 2 | 3 | 3 | 28 |
| NNT | 2 | 1 | 2 | 1 | |
| PNT | 18 | 1 | 1 | 2 | |
| Chemotherapy | | | | | |
| (–) | 31 | 1 | 3 | 7 | 42 |
| (+) | 44 | 9 | 8 | 18 | 79 |

[a]Subjects followed by the 'General Rule for Clinical and Pathological Studies on Renal Pelvic and Ureteral Cancer'. Grade, tumour grade; pT, depth of penetration; LY or V, lymphatic or venous invasion, M or N, distant organ or lymph node metastasis; growth, tumour growth pattern; NNT, non-papillary non-invasive type; PNT, papillary non-invasive type; NIT, non-papillary invasive type; PIT, papillary invasive type.

**Figure 2** The cumulative Kaplan–Meier survival curves of patients with carcinoma of renal pelvis and ureter in each group. There were significant differences between groups I and IV ($P < 0.001$) and between groups III and IV ($P < 0.001$) (**A**). With respect to tumour grade, of the 73 patients with low-grade TCC (grade 3 >, 3 < 2 or 1), there were significant differences between each group (between groups I and III, $P < 0.01$; I and IV, $P < 0.001$; III and IV, $P < 0.001$) (**B**). Of the 48 patients with high-grade TCCs (grade 3, 3 <), there was also a significant difference between these two groups ($P < 0.05$) (**C**)

non-cancerous tissues, including the epithelium, stroma adjacent to carcinoma and infiltrating inflammatory cells.

### Mirror section analysis of cyclin E and p53 immunopositivity

In the present study, there was good correlation for the expression between cyclin E and p53 protein in TCCs. Approximately 69% of cyclin E-positive TCCs (69.4%; 25 out of 36) also displayed positive reaction for p53, and there was a statistically significant correlation for the expression between these two proteins ($P < 0.05$). The mirror section technique revealed identical cancer cell nuclei that were positive for both cyclin E and p53 in all five tumours examined (Figure 1A and 1B).

In a previous study, we showed good correlation between p53 immunoreactivity and the presence of *p53* gene mutations concentrated on exons 4–9 in 26 cases (Furihata et al, 1995*b*). In the present study, 5 of these 26 tumours, including four tumours with both *p53* gene point mutations and protein overexpression, showed cyclin E immunoreactivity in the nucleus.

### Statistical analysis

The patients were divided into the following four groups on the basis of the pattern of tumour cell positivity for cyclin E and/or p53; group I, 75 cases (62.0%), cyclin E⁻/p53⁻, group II, ten cases (8.3%), cyclin E⁻/p53⁺; group III, 11 cases (9.0%), cyclin E⁺/p53⁻; and group IV, 25 cases (20.7%), cyclin E⁺/p53⁺. Table 2 shows the relationships between the rates of detection of overexpressed cyclin E and/or p53 in primary tumours and clinical or pathological features. The relationship of group IV with high nuclear grade ($P < 0.01$) was statistically significant. In contrast, no significant correlation was detected between cyclin E or p53 overexpression and the other clinical and pathological parameters (sex, age, tumour grade, depth of penetration, lymphatic or venous invasion, distant organ or lymph node metastasis, tumour growth patterns and treatment with chemotherapy). No significant correlation was detected in the other three groups with any of the clinicopathological factors analysed.

### Association between cyclin E and/or p53 overexpression and prognosis

Figure 2A shows the Kaplan–Meier survival curves based on a simultaneous comparison of the four groups of this cohort. The post-operative 10-year survival rate of group I (cyclin E⁻/p53⁻) was 67.7%, while that of group IV (cyclin E⁺/p53⁺) was 15.2%. There was a significant difference between these two groups ($P < 0.001$). The post-operative 9-year survival rate of group III (cyclin E⁺/p53⁻) was 88.2%, while that of group IV was 15.2%. There was also a significant difference between these groups ($P < 0.001$). In contrast, there was no significant difference in the survival rate between group I and group II (cyclin E⁻/p53⁺) or group III.

With respect to tumour grade, of the 73 patients with low-grade TCC (grade 3 >, 3 < 2 or 1), the post-operative 5-year survival rates of the groups I, III and IV were 74.8%, 97.6% and 13.8% respectively. There were significant differences between each group (between groups I and III, $P < 0.01$, I and IV, $P < 0.001$, III and IV, $P < 0.001$) (Fig. 2B). Of the 48 patients with high-grade TCC (grade 3, 3 <), the post-operative 9-year survival rate of groups I and IV were 61.0% and 19.8%, respectively. There was also a significant difference between these two groups ($P < 0.05$) (Figure 2C).

To determine the most informative combination of independent factors for prognosis, the variables identified as statistically significant in predicting survival (tumour grading, depth of penetration, lymphatic or venous invasion, distant organ or lymph node metastasis, tumour growth pattern and cyclin E and/or p53 overexpression) were subjected to a multivariate analysis using Cox's stepwise proportional hazard model. Each of these factors was analysed to determine whether they had statistically significant effects on the survival rate or not. A stepwise selection of these

© Cancer Research Campaign 1998

**Table 3** Summary of the statistical analysis of prognostic factors using the Cox proportional hazard model

| Prognostic factors | Hazard ratio (95% confidence limit) | P value |
|---|---|---|
| Tumour grade | 0.18 (0.04–0.82) | < 0.05 |
| M or N[a] | 9.20 (3.74–22.63) | < 0.001 |
| cyclin E and p53 | 8.20 (3.06–21.96) | < 0.001 |

[a]Distant organ or lymph node metastasis.

factors was made, based on the relative magnitude of their contribution to survival. As shown in Table 3, these analyses demonstrated that the most important factor affecting survival was the distant organ or lymph node metastasis ($P < 0.001$). Moreover, simultaneous overexpression of both cyclin E and p53 was also an important factor affecting survival ($P < 0.001$), as well as tumour grade ($P < 0.05$).

### DISCUSSION

We directly studied the relevance of cyclin derangement to in vivo conditions using immunohistochemistry assessing and analysing the expression of both cyclin E and p53 proteins in tumour samples from patients with TCCs. This immunohistochemical study using antibodies to cyclin E and p53 was optimally designed for precise measurement of the expression rates of these two proteins and their expression patterns in individual tumour cells, and this technique may be suitable for screening. In the present study, 29.8% (36 out of 121) of the tumour samples showed positive immunoreaction with cyclin E antibody, which was predominantly revealed in the nuclei of cancer cells. We also detected the overexpression of p53 protein in 35 TCCs, 25 of which showed simultaneous positivity for cyclin E. In addition, the simultaneous detection of cyclin E and p53 protein overexpression was demonstrated in identical cancer cell nuclei of TCCs. These findings suggest that both cyclin E and p53 protein overexpression frequently coexist in renal pelvic and ureteral TCCs.

The present study with urothelial TCC also revealed an interesting and significant relationship between cyclin E and p53 protein accumulation, demonstrating that the frequency of tumours with simultaneous alterations in both cyclin E and p53 protein expressions significantly increased along with the degree of tumour grade ($P < 0.01$), revealing poorer prognosis ($P < 0.001$). Keyomarsi et al (1994) demonstrated that cyclin E alterations become progressively worse with increasing stage and grade of the breast carcinoma. Recent reports have indicated that p53 abnormalities are commonly found in invasive and high-grade TCCs with poor prognosis (Furihata et al, 1993). In the renal pelvic TCCs, however, Terrell et al (1995) failed to show the significant association between p53 protein overexpression and established prognostic factors in 67 cases of TCCs. In the present study, we demonstrated additional evidence that the combined evaluation of cyclin E and p53 overexpression may provide prognostic information that is more accurate than the evaluation of p53 overexpression alone. Therefore, the immunohistochemical detection of both cyclin E and p53 protein is meaningful in the search for novel and potentially useful prognostic markers in renal pelvic and ureteral TCC.

Evidence has been accumulated to support the presence of a regulatory loop between cyclin E and p53 protein involving various Cdk inhibitors. In the molecular network of the normal cell cycle, cyclin–Cdk complexes, which induce progression of the cell cycle, are inactivated by $p21^{WAF1/CIP1}$, which is thought to be a downstream target of p53 and serves as an effector of cell cycle arrest in response to activation of p53 (Ei-Deiry et al, 1993; Harper et al, 1993). $p21^{WAF1/CIP1}$ inhibits different Cdks (Cdks 2, 4/6), working in conjunction with various cyclins (cyclin A, B, D, E) to alter the activity of key proteins controlling entry into the different phases ($G_1$, $G_1$/S, S, $G_2$/M phase) of the cell cycle (Ei-Deiry et al, 1993; Harper et al, 1993; Cross et al, 1995). In mammalian cells, the cyclin E–Cdk2 complex plays an important role in the $G_1$/S phases (Cordon-Cardo, 1995). In the present study of 26 tumours, which had already been examined for p53 mutations by comparing the immunohistochemical reactivity (Furihata et al, 1995*b*), cyclin E immunoreactivity was detected in five cyclin E-positive tumours, including four tumours with both *p53* gene mutations and accumulation of its products. Recently, Akama et al (1996) studied the relationship between *p53* mutations and the expression of $p21^{WAF1/CIP1}$ or cyclins in human gastric cancer cell lines and showed the correlation between *p53* gene mutations and very low or undetectable levels of $p21^{WAF1/CIP1}$ mRNA expression. In addition, an inverse correlation was simultaneously shown between the level of $p21^{WAF1/CIP1}$ and cyclin E mRNA in the same cell lines examined (Akama et al, 1996). Elbendary et al (1996) also showed that mutation of the *p53* gene was associated with decreased $p21^{WAF1/CIP1}$ expression in human ovarian cancers. A recent immunohistochemical study in human breast carcinoma showed that most tumours with *p53* gene mutations revealed low to absent $p21^{WAF1/CIP1}$ immunoreactivity (Barbareschi et al, 1996). Although the effects of unscheduled overexpression of cyclin E intimately related to p53 alteration in the development of TCC have not yet been explored, it is interesting to speculate that alteration of the *p53* gene, leading to accumulation of its product, cannot induce activation of Cdk inhibitors, such as $p21^{WAF1/CIP1}$, and thus additional cancer-promoting genetic alterations through $G_1$/S phases of the cell cycle, including those related to aberrant cyclin E gene transactivation leading to post-transcriptional overexpression of its product, may follow. On the other hand, recent studies have revealed a p53-independent pathway of $p21^{WAF1/CIP1}$ gene activation in primary embryo fibroblasts (Michiele et al, 1994), human promyelocytic HL-60 leukaemia cells (Jiang et al, 1994) and human breast cancer cells (Sheikh et al, 1994). Although the molecular basis for the activation of Cdk inhibitors, including the $p21^{WAF1/CIP1}$ gene, is still indefinite, our findings that 11 cases were immunohistochemically positive for cyclin E but negative for p53 may also suggest in part that p53 is not the sole regulator of the upstream pathway in the cyclin E gene expression which is inhibited by activating the $p21^{WAF1/CIP1}$ gene. With respect to cyclin E abnormality in tumorigenesis, little direct experimental evidence exists supporting the correlation between cyclin E gene alterations, such as gene amplification or transformation, and overexpression of its product (Keyomarsi and Pardee, 1993; Keyomarsi et al, 1995; Courjal et al, 1996). In order to determine whether both p53 and cyclin E alterations enhance genetic changes during the progression of the renal pelvic and ureteral TCC, the relationship between *p53* gene abnormalities and subsequent transactivation or genetic changes of each cyclin gene, especially of cyclin E, which promotes an advance in the cell cycle of tumour cells, should be further examined.

In conclusion, this is the first report of frequent cyclin E protein overexpression in association with p53 alteration in a large series of primary human renal pelvic and ureteral TCCs. In addition, the

© Cancer Research Campaign 1998

present study also demonstrates that simultaneous overexpression of both cyclin E and p53 protein may be important prognostic indicators and may be potentially useful for assessing tumour aggressiveness. Further comprehensive studies using excised human carcinoma tissue samples in greater numbers, including measurement of DNA and/or RNA levels, will be required to confirm these results.

### ACKNOWLEDGEMENTS

We are grateful to Dr T Moriki, Department of Clinical Laboratories, Kochi Medical School Hospital, for providing clinical materials, and Dr H Sasano, Department of Pathology, Tohoku University School of Medicine, for his comments. This study was supported in part by Grants-in Aid for Scientific Research© from the Ministry of Education, Science and Culture of Japan.

### REFERENCES

Akama Y, Yasui W, Kuniyasu H, Yokozaki H, Akagi M, Tahara H, Ishikawa T and Tahara E (1996) Genetic status and expression of the cyclin-dependent kinase inhibitors in human gastric carcinoma cell lines. *Jpn J Cancer Res* **87**: 824–830

Barbareschi M, Caffo O, Doglioni C, Marchetti A, Buttitta F, Leek R, Bevilacqua G, Dalla Palma P and Harris AL (1996) p21 Waf 1 immunohistochemical expression in breast carcinoma: correlation with clinic-pathologic data, ER status, MIB 1 expression, p53 gene and protein alteration and relapse free survival. *Br J Cancer* **74**: 208–215

Betticher D, Thatcher N, Altermatt HJ, Hoban P, Ryder WDJ and Heighway J (1995) Alternate splicing produces a novel cyclin D1 transcript. *Oncogene* **11**: 1005–1011

Buckley MF, Sweeney KJ, Hamilton JA, Sini RL, Manning DL, Nicholson RI, deFazio A, Watts CKW, Musgrove EA and Sutherland RL (1993) Expression and amplification of cyclin genes in human breast cancer. *Oncogene* **8**: 2127–2133

Cong J, Ardelt B, Traganos F and Darzynkiewicz Z (1994) Unscheduled expression of cyclin B1 and cyclin E in several leukemic and solid tumour cell lines. *Cancer Res* **54**: 4285–4288

Cordon-Cardo C (1995) Mutation of cell cycle regulators. Biological and clinical implications for human neoplasia. *Am J Pathol* **147**: 545–560

Courjal F, Louason G, Speiser P, Katsaros D, Zeillinger R and Theillet C (1996) Cyclin gene amplification and overexpression in breast and ovarian cancer: evidence for the selection of cyclin D1 in breast and cyclin E in ovarian tumours. *Int J Cancer* (*Pred Oncol*) **69**: 247–253

Cross SM, Sanchez CA, Morgan CA, Schimke MK, Ramel S, Idzerda RL, Raskind WH and Reid BJ (1995) A p53-dependent mouse spindle checkpoint. *Science* **267**: 1353–1356

Elbendary AA, Cirisano FD, Evans AC, Davis JPL, Iglehart JD, Marks JR and Berchuck A (1996) Relationship between p21 expression and mutation of the p53 tumour suppressor gene in normal and malignant ovarian epithelial cells. *Clin Cancer Res* **2**: 1571–1575

El-Deiry WS, Tokino T, Velculescu VE, Levy DB, Parsons R, Trent JM, Lin D, Mercer E, Kinzler KW and Vogelstein B (1993) WAF1, a potential mediator of p53 tumour suppression. *Cell* **75**: 817–825

Elledge SJ and Spottswood MR (1991) A new human p34 protein kinase, CDK2, identified by complimentation of a CDC28 mutation in *Saccharomyces cerevisiae*, is a homolog of Xenopus Eg1. *EMBO J* **10**: 2653–2659

Furihata M, Inoue K, Ohtsuki Y, Hashimoto H, Terao N and Fujita Y (1993) High-risk human papillomavirus infections and overexpression of p53 protein as prognostic indicators in transitional cell carcinoma of the urinary bladder. *Cancer Res* **53**: 4823–4827

Furihata M, Sonobe H and Ohtsuki Y (1995*a*) The aberrant p53 protein (review). *Int J Oncol* **6**: 1209–1226

Furihata M, Yamasaki I, Ohtsuki Y, Sonobe H, Morioka M, Yamamoto A, Terao N, Kuwahara M and Fujisaki N (1995*b*) p53 and human papillomavirus DNA in renal pelvic and ureteral carcinoma including dysplastic lesions. *Int J Cancer* (*Pred Oncol*) **64**: 298–303

Furihata M, Sonobe H, Ohtsuki Y, Yamashita M, Morioka M, Yamamoto A, Terao N, Kuwahara M and Fujisaki N (1996) Detection of p53 and bcl-2 protein in carcinoma of the renal pelvis and ureter including dysplasia. *J Pathol* **178**: 133–139

Gillett C, Ranti V, Smith R, Fisher C, Bartek J, Dickson C, Barnes D and Peters G (1994) Amplification and overexpression of cyclin D1 in breast cancer detected by immunohistochemical staining. *Cancer Res* **54**: 1812–1817

Harper JW, Adami GR, Wei N, Keyomarsi K and Elledge SJ (1993) The p21 Cdk-interacting protein Cip1 is a potent inhibitor of G1 cyclin-dependent kinases. *Cell* **75**: 805–816

Hunter T and Pines J (1991) Cyclins and cancer. A review. *Cell* **66**: 1071–1074

Ishikawa T, Furihata M, Ohtsuki Y, Murakami H, Inoue A and Ogoshi S (1998) Cyclin D1 overexpression related to retinoblastoma protein expression as a prognostic marker in human esophageal squamous cell carcinoma. *Br J Cancer* 77: 92–97

Jares P, Fernandez PL, Campo E, Nadal A, Bosch F, Aiza G, Nayach I, Traserra J and Cardesa A (1994) PRAD-1/Cyclin D1 gene amplification correlates with messenger RNA overexpression and tumour progression in human laryngeal carcinomas. *Cancer Res* **54**: 4813–4817

Jiang W, Zhang YJ, Kahn SM, Hollstein MC, Santella RM, Lu SH, Harris CC, Montesano R and Weinstein IB (1993) Altered expression of cyclin D1 and retinoblastoma genes in human esophageal cancer. *Proc Natl Acad Sci USA* **90**: 9026–9030

Jiang H, Lin J, Su Z, Collart FR, Huberman E and Fisher PB (1994) Induction of differentiation in human promyelocytic HL-60 leukemia cells activates p21, WAF1/CIP1, expression in the absence of p53. *Oncogene* **9**: 3397–3406

Keyomarsi K and Pardee AB (1993) Redundant cyclin overexpression and gene amplification in breast cancer cells. *Proc Natl Acad Sci USA* **90**: 1112–1116

Keyomarsi K, O'Leary N, Molnar G, Lees E, Fingert HJ and Pardee AB (1994) Cyclin E, a potential prognostic marker for breast cancer. *Cancer Res* **54**: 380–385

Keyomarsi K, Conte Jr D, Toyofuku W and Fox MP (1995) Deregulation of cyclin E in breast cancer. *Oncogene* **11**: 941–950

Leach FS, Elledge SJ, Sherr CJ, Willson JKV, Markowitz S, Kinzler KW and Vogelstein B (1993) Amplification of cyclin genes in colorectal carcinomas. *Cancer Res* **53**: 1986–1989

Michalides R, van Veelen N, Hart A, Loftus B, Wienjens E and Balm A (1995) Overexpression of cyclin D1 correlates with recurrence in a group of forty-seven operable squamous cell carcinomas of the head and neck. *Cancer Res* **55**: 975–978

Michiele P, Chedid M, Lin D, Mercer WE and Givol D (1994) Induction of WAF1/CIP1 by a p53-independent pathway. *Cancer Res* **54**: 3391–3395

Pines J and Hunter T (1989) Isolation of a human cyclin cDNA: evidence for cyclin mRNA and protein regulation in the cell cycle and for interaction with $p34^{cdc2}$. *Cell* **58**: 833–846

Rosenblatt J, Gu Y and Morgan DO (1992) Human cyclin-dependent kinase 2 is activated during the S and G2 phase of the cell cycle and associates with cyclin A. *Proc Natl Acad Sci USA* **89**: 2824–2828

Sheikh MS, Li XS, Chen JC, Shao ZM, Ordonez JV and Fontana JA (1994) Mechanisms of regulation of WAF1/Cip1 gene expression in human breast carcinoma: role of p53-dependent and independent signal transduction pathway. *Oncogene* **9**: 3407–3415

Sidransky D, Eschenbach AV, Tsai YC, Jones P, Summerhayes I, Marshall F, Paul M, Green P, Hamilton SR, Frost P and Vogelstein B (1991) Identification of p53 gene mutations in bladder cancers and urine samples. *Science* **252**: 706–709

Terrell RB, Cheville JC, See WA and Cohen MB (1995) Histopathological features and p53 nuclear protein staining as predictors of survival and tumour recurrence in patients with transitional cell carcinoma of the renal pelvis. *J Urol* **154**: 1342–1347

Tsai LH, Harlow E and Meyerson M (1991) Isolation of the human cdk2 gene that encodes the cyclin A- and adenovirus E1A-associated p33 kinase. *Nature* **353**: 174–177

Yasui W, Kuniyasu H, Yokozaki H, Semba S, Shimamoto F and Tahara E (1996) Expression of cyclin E in colorectal adenomas and adenocarcinomas: correlation with expression of Ki-67 antigen and p53 protein. *Virchow Arch* **429**: 13–19

© Cancer Research Campaign 1998